# Mannitol and renal graft injury in patients undergoing deceased donor renal transplantation – a randomized controlled clinical trial

**DOI:** 10.1186/s12882-020-01961-z

**Published:** 2020-07-28

**Authors:** Christian Reiterer, Karin Hu, Samir Sljivic, Markus Falkner von Sonnenburg, Edith Fleischmann, Alexander Kainz, Barbara Kabon

**Affiliations:** 1 Department of Anaesthesia, Intensive Care Medicine and Pain Medicine, Medical University of Vienna, Spitalgasse 23, 1090 Vienna, Austria; 2 Clinical Department of Nephrology and Dialysis, Medical University of Vienna, Vienna, Austria

**Keywords:** Mannitol, Renal transplantation, I/R injury, Renoprotection, Biology-derived biomarker

## Abstract

**Background:**

Ischaemia/reperfusion (I/R) injury is associated with renal tissue damage during deceased donor renal transplantation. The effect of mannitol to reduce I/R injury during graft reperfusion in renal transplant recipients is based on weak evidence. We evaluated the effect of mannitol to reduce renal graft injury represented by 16 serum biomarkers, which are indicators for different important pathophysiological pathways. Our primary outcome were differences in biomarker concentrations between the mannitol and the placebo group 24 h after graft reperfusion. Additionally, we performed a linear mixed linear model to account biomarker concentrations before renal transplantation.

**Methods:**

Thirty-four patients undergoing deceased donor renal transplantation were randomly assigned to receive either 20% mannitol or 0.9% NaCl placebo solution before, during, and after graft reperfusion. Sixteen serum biomarkers (MMP1, CHI3L1, CCL2, MMP8, HGF, GH, FGF23, Tie2, VCAM1, TNFR1, IGFBP7, IL18, NGAL, Endostatin, CystC, KIM1) were measured preoperatively and 24 h after graft reperfusion using Luminex assays and ELISA.

**Results:**

Sixteen patients in each group were analysed. Tie2 differed 24 h after graft reperfusion between both groups (*p* = 0.011). Change of log2 transformed concentration levels over time differed significantly in four biomarkers (VCAM1,Endostatin, KIM1, GH; *p* = 0.007; *p* = 0.013; *p* = 0.004; *p* = 0.033; respectively) out of 16 between both groups.

**Conclusion:**

This study showed no effect of mannitol on I/R injury in patients undergoing deceased renal transplantation. Thus, we do not support the routinely use of mannitol to attenuate I/R injury.

**Trial registration:**

NCT02705573. Registered on 10th March 2016.

## Background

Renal transplantation is the treatment of choice for patients with end-stage-renal disease [1, 2]. Renal ischaemia and reperfusion (I/R) injury is one of the leading causes mediating acute kidney injury (AKI) in the native as well as in the transplanted kidney [3]. Re-oxygenation after ischaemia results in tissue injury due to apoptosis and necrosis, and activation of inflammatory pathways triggering innate immune responses [4].

Mannitol, an osmotic diuretic, is routinely used to improve renal function after deceased donor renal transplantation [5]. It increases renal blood flow, supposedly attenuates I/R injury during graft reperfusion and acts as a free radical scavenger [6]. Additionally, it promotes the release of prostaglandins in the kidney leading to vasodilatation and consequently to an increased urine flow in the renal tubulars [7]. However, these beneficial effects are only based on weak evidence and the renoprotective potency of mannitol during renal transplantation is still lacking.

Estimated glomerular filtration rate (eGFR) after transplantation based on serum creatinine (sCr) is mainly used for the clinical assessment of renal function. Even though, measurement of sCr is still the state of the art to estimate renal function, the sensitivity is low for detecting AKI of biopsy proven tubular injury [8, 9]. Therefore, in the last decade a major focus of research was to detect new molecular biomarkers (BM) involved in disease relevant pathophysiological pathways.

In the chronic disease, BM were identified following a systems biology approach, which is based on a silico–derived molecular model of diabetic kidney disease. A parsimonious set of BM covering multiple relevant pathways was validated in a large group of patients with type II diabetes and was able to improve the predictive power for eGFR loss on top of clinical covariates [10, 11].

Similarly, promising BM for AKI, delayed graft function and renal transplant outcome were identified [9, 12–16]. Consequently, we tested the intraoperative use of mannitol on a set of 16 BM representing different molecular processes involved in ischaemic kidney injury, inflammation and tubular damage.

## Methods

This prospective double-blinded, randomized trial was performed at the Department of Anaesthesia, Intensive Care Medicine and Pain Medicine and the Department of Nephrology and Dialysis, Medical University of Vienna, Vienna, Austria. The trial was approved by the local ethics committee of the Medical University of Vienna in 2014 (Chairman: J. Zezula, MD) (EK 2021/2014) and was registered at ClinicalTrials.gov (NCT02705573) and EudraCT (2014–005391-29) and conducted according to the Declaration of Helsinki and Good Clinical Practice.

Written informed consent was obtained from all patients. Patients with end-stage renal disease between 18 and 80 years of age undergoing deceased donor renal transplantation were included. Exclusion criterion was a known allergy to mannitol. All patients were hemodialysed shortly before renal transplantation.

Non-heart-beating donors were not included. Hypothermic machine perfusion of deceased donor kidneys was not performed.

### Randomization

Patient allocated to the mannitol group received a 20% mannitol solution in a dose of 5 mL/kg bodyweight (BW) (Concentration: 5 mL = 1 g) The placebo group received 0.9% NaCl solution in a dose of 5 mL/kg BW. The maximum dose of the study medication was restricted to 500 mL.

A bolus of 100 mL of the study solution was administered shortly before graft reperfusion. The remaining study solution was infused till the end of surgery. Randomization and blinding of the study drug were performed by the pharmacy. The patient, the attending anesthesiologist, and the research team were unaware of the group allocation.

Randomization sequence was created by an investigator with no clinical involvement in the trial using simple randomization procedures.

### Protocol

Anesthesia was induced with 2–3 μg kg^− 1^ BW fentanyl and 2–3 mg kg^− 1^ BW propofol. Muscle relaxation was performed at the discretion of the attending anesthesiologist. Narcotrend guided anesthesia was maintained with sevoflurane in 30% oxygen. Additional fentanyl was administered according to patient’s requirements. We kept end-tidal CO_2_ at near 4.7 kPa. Non-invasive blood pressure was measured in 5-min intervals. Normothermia was maintained with forced-air warming. According to clinical standards, all patients received a central venous line. Central venous blood gas samples were obtained hourly.

Fluid administration was esophageal Doppler guided (Cardio Q, Deltex Medical, Chichester, UK) according to a previous published algorithm [17]. A balanced crystalloid solution (Elomel isoton; Fresenius Kabi, Austria) was used for intraoperative fluid replacement therapy. All patients received a baseline infusion rate of 2 mL kgBW^− 1^ h^1^. We performed intraoperative goal-directed fluid management using esophageal Doppler monitoring (CardioQ; Deletex Medical, Chicester, UK). Our fluid management was based on the algorithm published by the Anesthesia Working Group of the ‘Enhanced Recovery after Surgery (ERAS) Society [17] and slightly modified.

We placed the esophageal Doppler probe after induction of anesthesia. After the characteristic Doppler signal was displayed, a fluid challenge of 250 mL was administered to assess stroke volume (SV) response. If the SV increased > 10% (i.e. fluid responder), a further fluid bolus was administered. This was repeated as often as no further increase of more than 10% in SV was detected. In fluid non-responders we treated coexisting hypotensive episodes, which were defined by a mean arterial pressure (MAP) < 70 mmHg in normotensive and < 80 mmHg in hypertensive patients, with vasopressor titration at the discretion of the attending anesthesiologist.

Hemodynamic parameters were re-evaluated at least every 15 min (or more frequently in case of significant hemodynamic changes, e.g. blood loss). When SV dropped more than 10%, we administered a further fluid bolus according to the above described algorithm.

Blood units were given as necessary. Transfusion trigger was a hemoglobin concentration of 7.0 mg dL^− 1^. However, if there was any clinical sign of organ hypoxemia (e.g. lactic acidosis) blood units were given earlier at the discretion of the attending anesthesiologist.

During the study period the use of diuretics was not allowed.

### Blood samples

Blood samples for BM measurements were taken in Z Serum Separator Clot Activator vacutainers (Greiner Bio-One, Austria). Blood was drawn shortly before induction of anesthesia and 24 h after graft reperfusion. Samples were allowed to clot for 30 min at room temperature before centrifuging at 1000 g for 10 min at room temperature. Serum was removed and stored at − 80 °C until further processing.

### Measurements

Demographic data, comorbidities, renal-replacement therapy, residual urinary output, long-term medication and preoperative laboratory values were obtained from patient’s medical records. Renal transplant specific data including arterial and venous vascular clamp times, intraoperative fluid requirements, blood loss, hemodynamic parameters and anesthesia specific management were recorded.

Donor and organ specific information including age, sex, laboratory values, diuresis, catecholamine support and cold and warm ischaemia time were provided by Eurotransplant.

Potassium, urinary output, creatinine, BUN and GFR were measured 24 h after graft reperfusion.

### Luminex serum measurements

Measurements of serum samples were carried out using three panels: Luminex 3-plex, Luminex 12-plex and ELISA. Quantikine ELISAs for human serum TIM-1/KIM-1/HAVCR (catalog no. DSKM100, R&D Systems, Minneapolis, MN) were used for measurement of KIM1 serum concentrations. Assays were processed according to the procedure guidelines provided. The optical density of each plate was measured within 30 min using a TriStar [2] LB 942 Modular Multimode Microplate Reader (Berthold Technologies, Bad Wildbad, Germany) set to 450 nm and wavelength correction set to 570 nm. The standard curve was generated using MikroWin2010 v5.21 software with a four-parameter logistic (4-PL) curve fit.

Three markers (Endostatin, CystC and NGAL) were measured using a Human Premixed Multi-Analyte Kit (catalog no.LXSAHM-03; R&D Systems). Samples were diluted 1:50 using Calibrator Diluent RD6–52 provided in the assay kit. The remaining twelve BM (MMP1, MMP8, HGF, GH, CHI3L1, TIE2, TNFR1, VCAM1, CCL2, FGF23, IL18 and IGFBP7) were measured using a Human Premixed Multi-Analyte Kit (catalog no.LXSAHM-12; R&D Systems) and a samples dilution of 1:2. Both assays were processed according to assay procedure provided by the manufacturer and measured on a Luminex 200 (Luminex Corporation, Austin, TX) with xPONENT software (version 3.1.971.0) set according to assay instructions.

Calibration and verification of the Luminex 200 was preformed once a week using Luminex 200 Performance Verification Kit (catalog no. 40–276, Merck Millipore, Billerica, MA) and Calibration Kit (catalog no. 40–275, Merck Millipore).

As no commercial quality controls (QC) were available for multiplex assays, Pooled Normal Human Plasma K3 EDTA (catalog no. IPLA-N-100 ml-K3 EDTA, Innovative Research, Novi, MI) was spiked with recombinant proteins (R&D Systems) and diluted to different concentrations to act as low, medium and high QC samples for the Luminex panel and the KIM1 ELISA. All samples were measured as two technical replicates. If replicate measurements display a coefficient of variation over 12%, the sample was remeasured. In addition, at least 10% of all samples were remeasured on a different plate to follow FDA and EMA guidelines which recommends incurred sample reanalysis with percentage difference between these samples below 20%. More than 66% of incurred sample reanalysis fulfilled this requirement and are therefore in concordance with FDA and EMA guidelines. Values out of quantifiable range were set to 0.5 and 1.5 times lower and upper quantification limits, respectively.

### Statistical analysis

Statistical analysis was performed using IBM SPSS Statistics (Version 25) and SAS 9.4 (Cary, NC). Normal distribution of the data was assessed with Kolmogorov-Smirnov test. Normally distributed data were presented as mean (standard deviation); not normally distributed data were given as median and percentile. Chi-square test was accomplished for comparing categorical variables. Interval variables between groups prior to transplantation were compared using a Mann-Whitney-U-test or a Student’s t-test. Post transplantation a mixed linear model adjusted for donor kidney pairs was used to assess our primary outcome: differences in serum BM concentrations between both study groups 24 h after graft reperfusion. Additionally, in order to take preoperative BM concentrations into account another mixed linear model for concentration changes over time for each of the sixteen biomarkers was performed. Concentrations were standardised for each group and biomarker (mean = 0, standard deviation = 1) to be able to compare the different models. In the model the dependent variable was the standardised concentration. Independent variables were treatment group and time point of measurement and their interaction. Additionally, we used the intercept and slope as random effects. (online Supplement).

A *p*-value < 0.05 was considered as statistically significant.

### Sample size consideration

Based on previous BM concentration measurements the standard deviation of the log2 transformed values in median (1st, 3rd quartile) is 0.86 (0.5–1.06). Therefore, in order to detect an effect size of 0.86 with a power of 80% using a two-sample t-test 17 patients in each study group were estimated to be sufficient to detect an significant difference in at leaset 50% of all BM.

## Results

**Fig. 1:**
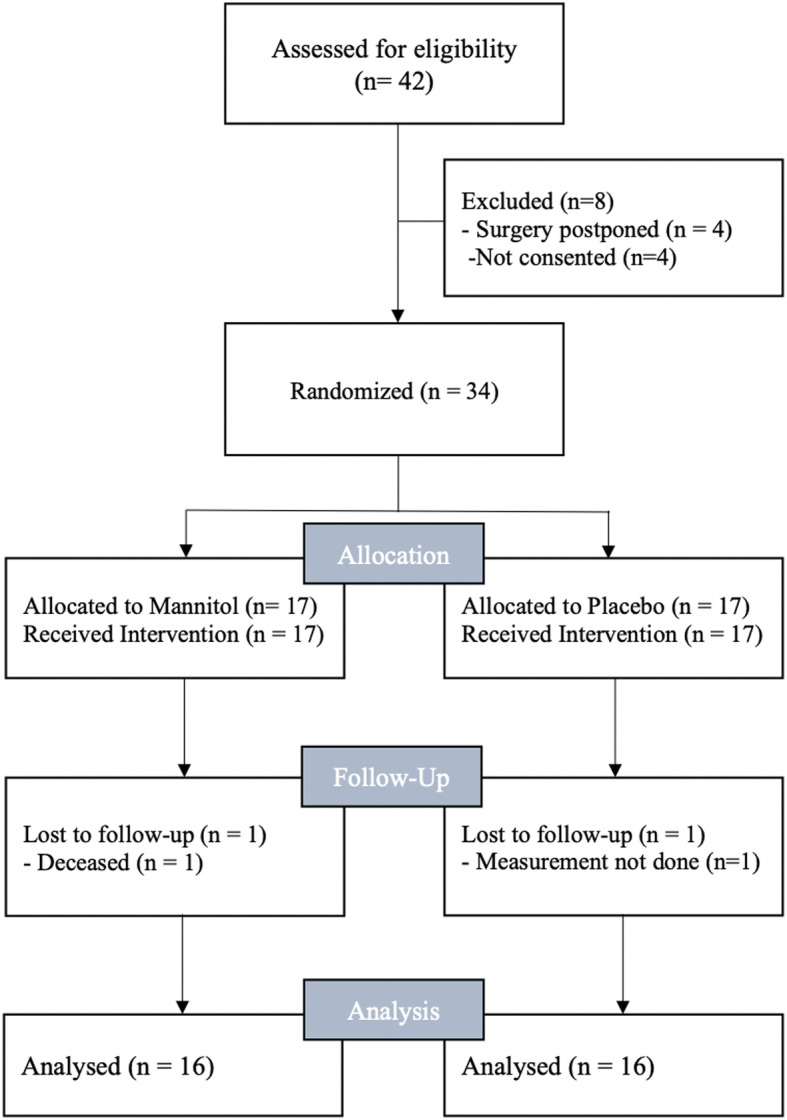
CONSORT 2010 patients flow chart

In total 34 patients with end-stage renal disease undergoing deceased donor renal transplantation were enrolled between January 2018 and July 2018. Seventeen patients received mannitol and 17 patients received placebo (Fig. 1). One patient in the mannitol group deceased within 24 h after transplantation; therefore, no follow-up measurement was available. Another patient from the placebo group was lost to follow up due to missing samples. Both patients were excluded from the analysis. Demographic and morphometric data are summarised in Table 1. Donor specific characteristics are available in the online supplement are shown in Table 2.

**Table 1:** Patient characteristics and preoperative data

	Mannitol (*n* = 16)		Placebo (*n* = 16)		*p* - Value
Age, yrs	62	[57, 71]	53	[45, 68]	*0.10*
Height, *cm*	167	± 10	170	± 15	*0.58*
Weight, *kg*	74	± 17	78	± 17	*0.67*
*Gender, no. (%)*					*0.64*
Men	9	(56)	9	(56)	
Women	7	(44)	7	(44)	
*Comorbidities, no. (%)*					
Hypertension	15	(94)	15	(94)	*1.00*
Diabetes	5	(31)	1	(6)	*0.13*
Insulin use	4	(25)	1	(6)	
Non-Insulin use	1	(6)	0	(0)	
Pulmonary	2	(13)	5	(31)	*0.39*
*Chronic Intermit. Dialysis, no. (%)*	11	(69)	13	(82)	*0.70*
*Peritoneal Dialysis, no. (%)*	5	(31)	3	(19)	*0.70*
GFR, *mL min*^*−1*^*1.73 m*^*−2*^	9.34	(2.7)	10.4	(3.4)	*0.36*
*Residual Urine Output (mL 24 h*^*− 1*^*)*	500	[0, 1000]	250	[0, 750]	0.54
*Patients with > 500 mL residual Urine volume, no. (%)*	8	(50)	9	(56)	*0.73*
*Long-Term Medication, no. (%)*					
Beta Blocker	13	(82)	12	(75)	*0.67*
ACE Inhibitors/AT1 Blocker	8	(50)	9	(56)	*0.72*
Diuretics	4	(25)	5	(31)	*0.69*
*Preoperative Laboratory*					
Hemoglobin, *g/dL*	11.8	[10.7, 12.5]	11.1	[10.4, 12.2]	*0.70*
Haematocrit, *%*	35.7	[32.5, 38.3]	33.2	[31.9, 37.4]	*0.67*
Creatinine, *mg/dL*	7.5	[5.5, 8,7]	8.3	[5.5, 9.8]	*0.47*
CRP, *mg/dL*	0.2	[0.1, 0.5]	0.2	[0.1, 0.7]	*0.64*

Summary statistics are presented as counts, percentages of patients, means ±SD, and median [25th percentile, 75th percentile]. All *P*-values are for unpaired Student’s *t*-tests, Mann-Whitney-U test or chi-square tests as appropriate. *ASA* American Society of Anesthesiologists physical status; *ACE* angiotensin converting enzyme; *AT1* angiotensin; *CRP* C-reactive protein

**Table 2:** Donor characteristics

	Mannitol (*n* = 16)		Placebo (*n* = 16)		*p*- Value
*Age*, *yrs*	55	(range 40, 75)	60	(range 41, 69)	0.70
*Sex*					0.47
Men, (*%*)	8	(50)	11	(69)	
Women, (*%*)	8	(50)	5	(31)	
*Laboratory*					
Creatinine *mg/dL*	0.7	[0.6, 1.0]	0.9	[0.7, 1.1]	0.06
BUN, *mg/dL*	25	[14, 42]	19	[17, 46]	0.83
*Diuresis*, *mL 24 h*^*−1*^	2730	[1860, 4560]	2800	[2155, 4540]	0.73
*Noradrenaline*, *μg kg*^*−1*^*min*^*− 1*^	0.15	[0.06, 0.19]	0.16	[0.04, 0.22]	0.81
*Duration of Ischaemia*					
Cold, *min*	774	[480, 1071]	768	[543, 1150]	0.78
Warm, *min*	45	[35, 53]	44	[30, 58]	0.91

Summary characteristics of donor specific data are presented as medians [25th percentile, 75th percentile]. All *P*-values are for unpaired Student’s *t*-tests or Mann-Whitney-U tests as appropriate. *BUN* blood urea nitrogen

Intraoperative characteristics including duration of anesthesia and surgery, fluid and hemodynamic data, anesthetic variables and central venous blood gas variables are shown in Table 3.

**Table 3:** Intraoperative parameters

	Mannitol (*n* = 16)		Placebo (*n* = 16)		*p*- Value
*Duration*					
Anesthesia, *hrs*	3.6	[3.1, 4.1]	3.8	[3.4, 4.3]	0.35
Surgery, *hrs*	2.6	[2.2, 3.2]	2.9	[2.4, 3.5]	0.55
Arterial Clamp, *min*	16	[10, 23]	24	[12, 29]	0.25
Venous Clamp, *min*	22	[16, 25]	20	[15, 25]	0.47
*Fluid & Hemodynamics*					
Total Fluid, *mL*	1.870	±817	1.823	±558	0.85
Blood Loss, *mL*	200	[100, 588]	175	[0, 375]	0.49
MAP, TWA *mmHg*	77	[74, 91]	79	[74, 82]	0.74
SV, *mL*	51	[43, 82]	64	[52, 72]	0.74
CO, *L min*^*−1*^	3.1	[2.5, 5.0]	4.3	[2.9, 4.7]	0.97
CVP, *mmHg*	13	±4	12	±4	0.47
*Anesthesia Variables*					
Propofol, *mg*	190	[123, 200]	200	[90, 230]	0.92
Fentanyl, *μg*	650	[500, 750]	550	[400, 800]	0.92
et Sevoflurane TWA, *%*	1.3	±0.5	1.5	±0.3	0.36
SpO_2_, *%*	99	[98, 99]	98	[96, 99]	0.15
Core, T°C	36.3	±0.4	36.4	±0.5	0.32
Phenylephrine					
No. of Patients, (*%*)	8	(50)	11	(69)	0.78
Cumulative Dose, *mg*	0.02	[0.0, 0.4]	0.08	[0.0, 0.19]	0.99
Noradrenaline					
No. of Patients, (*%*)	5	(32)	5	(32)	1.00
Cumulative Dose, *mg*	0.14	[0.00, 0.29]	0.11	[0.00, 0.20]	0.86
*Central Venous Blood Gas Analysis*					
pH	7.36	±0.1	7.37	±0.1	0.40
pCO_2,_*mmHg*	44	±7	46	±7	0.34
pO_2,_*mmHg*	49	[42; 56]	52	[46; 56]	0.38
Hb, *g/dL*	9.7	±1.4	9.6	±1.3	0.62
Na^+^, *mmol/L*	136	±3	138	±2	0.04
K^+^, *mmol/L*	4.7	±0.7	4.7	±0.4	0.93
Lactate, *mmol/L*	0.8	±0.2	0.8	±0.3	0.49

Summary characteristics of intraoperative measurements presented as means ±SD or medians [25th percentile, 75th percentile]. All *P*-values are for unpaired Student’s *t*-tests or Mann-Whitney-U tests as appropriate. *MAP* mean arterial pressure; *TWA* time weighted average; *SV* stroke volume; *FTc* corrected flow time; *CO* cardiac output, *CVP* central venous pressure, *pCO*_*2*_ partial pressure of carbon dioxide; *pO*_*2*_ partial pressure of oxygen; *BE* base excess; *Hb* hemoglobin

Routine parameters measured 24 h after graft reperfusion including potassium, urinary output, creatinine, BUN and GFR were compared between the two groups; none of those parameters displayed a significant difference (Table 4).

**Table 4:** Postoperative parameters within 24 h

	Mannitol (*n* = 16)		Placebo (*n* = 16)		*p* – Value
K^+^, *mmol/L*	4.6	±0.6	4.7	±1.4	0.284
Urinary Output, *mL*	1600	[690, 2750]	1125	[550, 2375]	0.678
Creatinine, *mgdL*	5.1	±2.5	5.7	±2.1	0.384
BUN, *mg/dL*	34.7	±11.9	33.9	±12.6	0.702
GFR, *mL min*^*−1*^*1.73 m*^*−2*^	11.8	±7.0	10.3	±5.4	0.436

Summary characteristics of postoperative measurements 24 h after graft reperfusion. Data are presented as means ±SD or medians [25th percentile, 75th percentile]. All *P*-values are for a mixed linear model adjusted for the correlated nature of paired donor kidneys. *BUN* blood urea nitrogen; *GFR* glomerular filtration rate

**Fig. 2:**
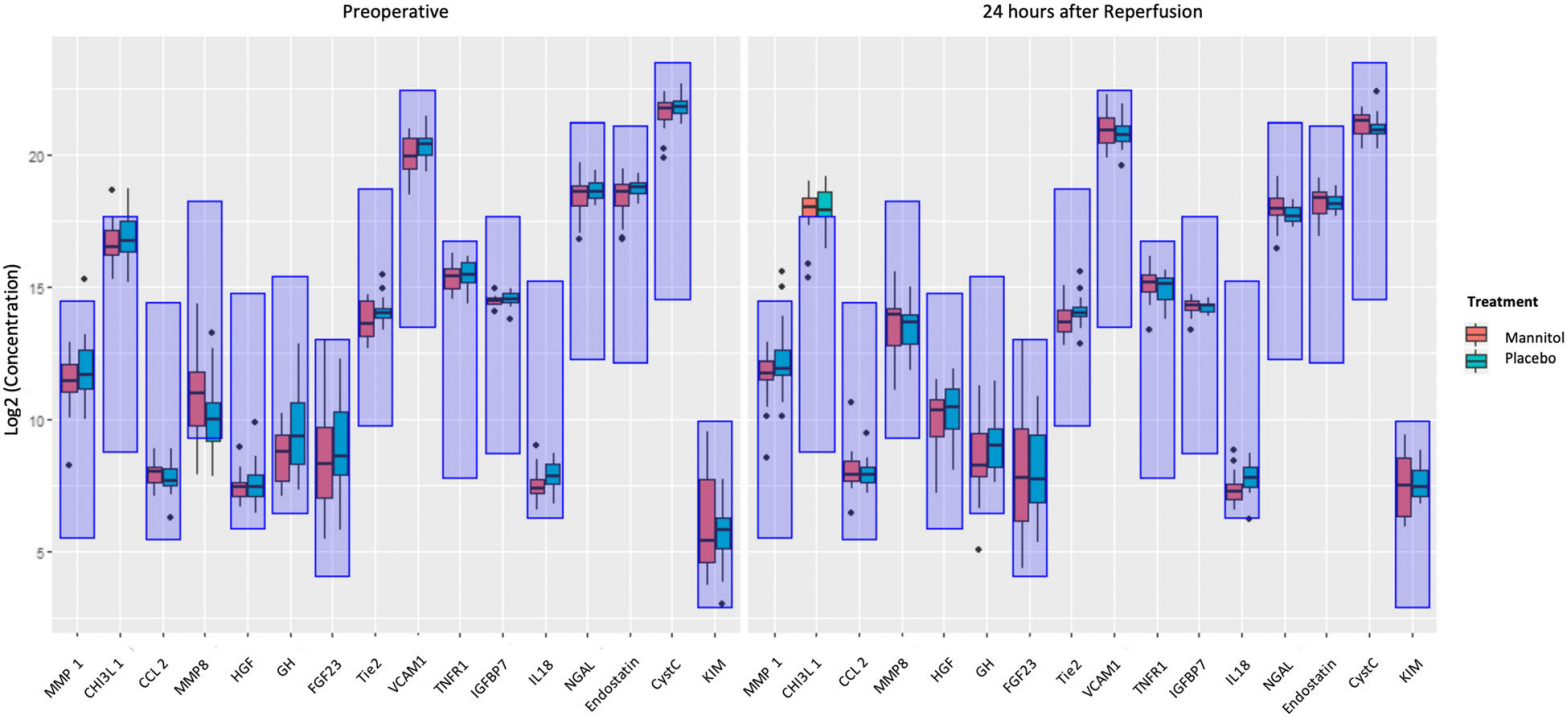
Concentration levels (log2 transformed) for all 16 biomarkers before transplantation and 24 h after graft reperfusion grouped by treatment. All concentrations are given in pg/mL. The quantifiable range of assays is indicated by areas shaded in blue

Concentration levels for all 16 BM before transplantation and 24 h after graft reperfusion grouped by treatment are illustrated in Fig. 2. Out of the 16 BM only Tie2 showed significant differences in concentration between the placebo group and the mannitol group 24 h after graft reperfusion (*p* = 0.011) (Table 5).

**Table 5:** Biology-derived Biomarkers

	Mannitol (*n* = 16)	Placebo (*n* = 16)	*p*-value
MMP1	3492 [3040, 4910]	3828 [3299, 6556]	0.360
CHI3L1	277,789 ± 119,127	290,668 ± 43,544	0.882
CCL2	244 [208, 314]	240 [196, 303]	0.711
MMP8	17,414 ± 11,898	13,001 ± 7340	0.126
HGF	1488 ± 847	1736 ± 1124	0.800
GH	286 [189, 598]	511 [292, 981]	0.387
FGF23	170 [67, 1673]	215 [116, 722]	0.183
Tie2	12,563 [9594, 18,103]	16,357 [14,596, 20,411]	0.011
VCAM1	2,057,315 [1,483,384, 3,106,136]	1,741,057 [1,422,526, 2,331,264]	0.354
TNFR1	36,465 ± 14,344	32,838 ± 10,274	0.272
IGFBP7	19,960 ± 4097	19,385 ± 2886	0.556
IL18	158 [130, 208]	221 [171, 304]	0.202
NGAL	252,684 [202,155, 340,047]	206,354 [182,217, 273,962]	0.091
Endostatin	314,261 ± 123,046	307,918 ± 74,018	0.981
CystC	2,405,546 [1,716,719, 2,976,273]	2,023,359 [1,794,789, 2,449,441]	0.994
KIM1	249 ± 197	227 ± 108	0.624

Testing for differences in BM concentrations 24 h after transplantation. Data are presented as means ±SD or medians [25th percentile, 75th percentile]. All concentrations are given in pg/mL A mixed linear model adjusted for paired donor kidneys was applied

In order to take BM concentrations before transplantation into account, linear mixed models were applied for changes in BM concentrations pre transplantation and 24 h after graft reperfusion. Change of log2 transformed concentration levels over time differed significantly between the groups in four out of 16 biomarkers only (online Supplement, eAppendix 1, Table S1). In the mannitol group there was a higher increase of VCAM1 than the placebo group (*p* = 0.007). On the contrary, KIM1 increased significantly less in patients receiving mannitol as compared to those receiving placebo (*p* = 0.004). Endostatin and GH concentrations decreased significantly less in the mannitol group as compared to the placebo groups (*p* = 0.013 and *p* = 0.033, respectively).

## Discussion

This study tested the efficiency of mannitol to reduce I/R injury during kidney transplantations. eGFR is the standard clinical parameter to evaluate kidney function but lacks sensitivity for small changes in the filtration rate and is not able to reflect pathophysiological processes [18]. Therefore, we used a set of 16 serum BM representing different pathways involved in ischaemic injury and inflammation in patients with end-stage renal disease undergoing deceased donor renal transplantation.

Although mannitol has been used for over 30 years in clinical practice, there is only weak evidence of its beneficial effects [19]. Renoprotection e.g. superoxide radical scavenging, increased renal blood flow and/or less tubular necrosis were mostly found based on preclinical animal studies [20]. Specifically, data for the renoprotective potency during renal transplantation are still lacking. Data from clinical studies, mostly performed in 1980’s revealed that mannitol reduced the incidence of acute tubular necrosis and decreased the number of dialysis required after transplantation. However no beneficial long-term effect could be observed in those studies [21–23].

A retrospective analysis based on an e-survey in 2011 showed that the use of diuretics during renal transplantation does not improve renal graft survival [24]. Although, of the lack of of high quality studies mannitol is still routinely used in some high-volume transplantation centres throughout renal transplantation [5].

It seems unlikely that mannitol affects long-term graft function; we thus focused on the immediate postoperative period. eGFR is still considered to be the best clinical marker to predict renal graft function. Direct measurements, however, are mostly infeasible; rather equitation based on demographic parameters, which’s accuracy to reflect real renal function in kidney transplant patients are limited [25], are used to estimate eGFR. Therefore, we measured a panel of promising BM to assess the renoprotective effect of mannitol.

We expected to see effects of mannitol reflected in the BM profile of the study patients. However, out of 16 BM only Tie2 was significantly different between the mannitol and the placebo group 24 h after transplantation. If BM concentrations before transplantation are taken into account only four BM (KIM1, Endostatin, GH and VCAM1) showed significant different changes in concentration over time.

The Angiopoietin(Ang)/Tie2 system is an important regulator for vessel stabilization and destabilization, and further to cope with injuries caused by ischemia and inflammation through binding of Ang1 to Tie2 as well as interactions of Ang2 as a context-dependent Tie2 antagonist. Shedding of Tie2 due to proteolytic cleavage occure upon various stimuli such as vascular endothelial growth factor (VEGF) and result in blocking of downstream signaling as soluble Tie2 acts as a competitive ligand for Ang1 and Ang2 [26]. A higher sTie serum level has been seen in serveral clinical conditions such as inflammation and cardiovascular disease [27, 28]. Additionally, sTie to has been shown to be correlated to VEGF which is dramatically upregulated under hypoxic circumstances [29]. Though, the exact mechanism of sTie2 in ischemia is not well understood it plays an important role in the regulation of angiogenesis and vascular inflammation. In our study Tie2 concentrations 24 h after transplantation were significantly higher in the placebo group. But, after taking pre transplantation concentrations into account by comparing the changes of concentrationen over time Tie2 was no longer significantly different between study groups.

KIM1, NGAL and IGFBP7 are specific biomarkers to detect renal tubular injury [30, 31]. KIM1 increased significantly less in the mannitol group over 24 h after graft reperfusion. However, there were no differences in NGAL and IGFBP7 in both groups over time. As concentrations of these three BM measured 24 h after reperfusion did not differ significantly between both groups, a protective effect of mannitol on tubular damages remains still questionable.

I/R injury is associated with inflammation and plays a crucial role in tissue damage and immune cell recruitment. One important protein is VCAM1, which is triggered by cytokines leading to endothelial cell-leukocytes adhesion and trans-endothelial migration into inflamed tissue. In vitro studies confirmed VCAM1 as a major contributor for leukocyte recruitment and consequently tissue damage during I/R injury [32]. Upregulation of VCAM1 correlates with structural tubular damage and fibrotic changes [33]. In our study population VCAM1 significantly increased over time in the mannitol group but no difference could be detected in VCAM1 concentrations 24 h after graft reperfusion.

Endostatin is the c-terminal fragment of collagen XVII, which is expressed in the membranes of glomerular, tubular epithelium, and vascular endothelium cells [34, 35]. Endostatin increases significantly in patients with AKI [36]. In our study population there was a decrease of Endostatin 24 h after transplantation, however there was no difference between both study groups.

IL18 is a proinflammatory cytokine and is a mediator and biomarker of ischaemic tissue damage [37]. Tubular epithelial cells express NGAL in response to tubulointerstitial cell injury which is mainly released by leucocytes, the loop of Henle and the collecting ducts [38]. NGAL is significantly higher in patients with delayed graft dysfunction after transplantation. Cystatin C is a promising serum BM for all-cause AKI with a pooled sensitivity, specificity and AUROC of 0.82, 0.82 and 0.89, respectively [39]. FGF23 is an early predictor and fast responding biomarker for AKI [40] and is significantly higher in patients developing AKI after cardiac surgery and ICU patients [41].

There are few BM which display statistically significant differences in concentration changes over time or in concentrations 24 h post transplantation but the BM panel as a whole provides little evidence for differences in the study groups. Additionally, we measured routine clinical parameters such as creatinine, urinary output and potassium. There was no difference in those parameters, which further underlines the lack of efficacy of mannitol in patients undergoing deceased renal transplantation.

Adequate intraoperative hydration during renal transplantation is associated with a lower incidence of delayed graft function [42, 43]. Thus, the strength of our study is esophageal Doppler guided fluid management and stroke volume optimization in all of our patients. Therefore, we can exclude differences in fluid volume status as a potential confounder. Additionally, protein measurements were carried out strictly according to protocol – samples were stored at − 80 °C and never thawn prior to measurements. Rigorous QC control according to FDA and EMA guidelines ensure accurate and reproducible results.

Since the effect of manntol begins within minutes after administrationit seems unlikely that a single dose of mannitol might improve renal function in the long-term. Therefore, we focused on the effect within the 24 h after graft reperfusion evaluated with our set of 16 BM. Because of our small sample size we were not able to draw any conclusions regarding postoperativ long-term renal graft function e.g. requirement of dialysis. Furthermore, we did not assess our biomarkers in healthy patients without any renal dysfunction which might have provided valuable addintional physiological renal background information.

## Conclusion

Few of the measured BM showed statistically differences in concentrations 24 h after transplantation between the mannitol and the placebo group as well as differences in concentration changes over time. Nevertheless the whole BM panel displayed limited evidence for the supposedly renoprotective effect of mannitol in patients exposed to reperfusion injury after deceased renal transplantation. We thus do not support the routine use of mannitol during graft reperfusion. This study awaits confirmation in an outcome trial.

## Supplementary information

**Additional file 1.**

## Data Availability

The datasets used and/or analyzed during the current study are available from the corresponding author on reasonable request. Corresponding author: edith.fleischmann@meduniwien.ac.at

## Abbreviations

MMP1: Matrix metalloproteinase-9
CCL2: Chemokine (C-C motif) ligand-2
MMP8: Matrix metalloproteinase-8
GH: Growth hormone
FGF23: Fibroblast growth factor-23
Tie2: Tyrosine Kinase-2
IGFBP7: Insulin Like Growth Factor Binding Protein-7
Endostatin: Endostatin
KIM1: Kidney Injury Molecule-1
CHI3L1: Chitinase-3-like protein-1
HGF: Hepatocyte growth factor
VCAM1: Vascular cell adhesion protein-1
TNFR1: Tumor necrosis factor receptor-1
IL18: Interleukin-18
NGAL: Neutrophil gelatinase-associated lipocalin
CystC: Cystatin C
