# Supplementary material for: Mannitol and renal graft injury in patients undergoing deceased donor renal transplantation – a randomized controlled clinical trial
Source: BMC Nephrol. 2020 Jul 28;21:307. doi: 10.1186/s12882-020-01961-z (PMC7388216; doi:10.1186/s12882-020-01961-z)
Supplement: Supplementary file 1 — Additional file 1. [file 12882_2020_1961_MOESM1_ESM.docx]

**Supplementary Online Content**

Christian Reiterer, MD; Karin Hu, MD; Samir Sljivic, MD; Markus Falkner von Sonnenburg, MD; Edith Fleischmann, MD; Alexander Kainz, PhD; Barbara Kabon, MD

**eAppendix 1:** Mixed linear model analysis

This supplementary material has been provided by the authors to give readers additional information about their work.

**eAppendix 1**

Mixed linear model for concentration changes over time adjusted for paired donor kidneys. Estimate time describes change of BM concentrations independent of treatment; Estimate time*treatment shows the change difference of the mannitol groups compared to the placebo group over time.

**Table S1**

|  | **Effect time** | | **Effect time * treatment** | |
| --- | --- | --- | --- | --- |
| **Parameter** | **Estimate** | **p-value** | **Estimate** | **p-value** |
| MMP1 | 0.235 | 0.361 | 0.041 | 0.808 |
| CCL2 | 0.520 | 0.134 | -0.846 | 0.116 |
| MMP8 | 1.081 | <0.001 | 0.281 | 0.381 |
| GH | -0.765 | 0.009 | 0.756 | **0.033** |
| FGF23 | -0.254 | 0.061 | 0.239 | 0.218 |
| Tie2 | -0.047 | 0.951 | 0.012 | 0.951 |
| IGFBP7 | -1.180 | <0.001 | 0.427 | 0.277 |
| Endostatin | -1.194 | <0.001 | 0.828 | **0.013** |
| KIM1 | 0.872 | <0.001 | -0.643 | **0.004** |
| CHI3L1 | 1.066 | <0.001 | 0.013 | 0.969 |
| HGF | 1.507 | <0.001 | -0.212 | 0.549 |
| VCAM1 | 0.521 | 0.010 | 0.773 | **0.007** |
| TNFR1 | -0.985 | <0.001 | 0.508 | 0.099 |
| IL18 | -0.048 | 0.042 | -0.048 | 0.696 |
| NGAL | -1.240 | <0.001 | 0.527 | 0.112 |
| CystC | -1.278 | <0.001 | 0.622 | 0.125 |
